# The human equilibrative nucleoside transporter 1 mediates *in vitro* cytarabine sensitivity in childhood acute myeloid leukaemia

**DOI:** 10.1038/sj.bjc.6602881

**Published:** 2005-12-06

**Authors:** I Hubeek, R W Stam, G J Peters, R Broekhuizen, J P P Meijerink, E R van Wering, B E S Gibson, U Creutzig, C M Zwaan, J Cloos, D J Kuik, R Pieters, G J L Kaspers

**Affiliations:** 1 Department of Pediatric Hematology/Oncology, VU University Medical Center, De Boelelaan 1117, Postbus 7057, 1007 MB, Amsterdam, The Netherlands; 2 Department of Medical Oncology, VU University Medical Center, De Boelelaan 1117, Postbus 7057, 1007 MB, Amsterdam, The Netherlands; 3 Department of Clinical Epidemiology and Biostatistics, VU University Medical Center, De Boelelaan 1117, Postbus 7057, 1007 MB, Amsterdam, The Netherlands; 4 Department of Pediatric Oncology/Hematology, Erasmus MC/Sophia Children's Hospital, Rotterdam, The Netherlands; 5 The Dutch Childhood Oncology Group (DCOG), The Hague, The Netherlands; 6 MRC Childhood Leukaemia Working Party, UK; 7 AML BFM-Study Group, Münster, Germany

**Keywords:** childhood acute myeloid leukaemia, deoxynucleoside analogues, cytarabine, hENT1

## Abstract

Cytarabine (ara-C) is the most effective agent for the treatment of acute myeloid leukaemia (AML). Aberrant expression of enzymes involved in the transport/metabolism of ara-C could explain drug resistance. We determined mRNA expression of these factors using quantitative-real-time-PCR in leukemic blasts from children diagnosed with *de novo* AML. Expression of the inactivating enzyme pyrimidine nucleotidase-I (PN-I) was 1.8-fold lower in FAB-M5 as compared to FAB-M1/2 (*P*=0.007). *In vitro* sensitivity to deoxynucleoside analogues was determined using the MTT-assay. Human equilibrative nucleoside transporter-1 (*hENT1)* mRNA expression and ara-C sensitivity were significantly correlated (*r*_p_=−0.46; *P*=0.001), with three-fold lower *hENT1* mRNA levels in resistant patients (*P*=0.003). *hENT1* mRNA expression also seemed to correlate inversely with the LC_50_ values of cladribine (*r*_p_=−0.30; *P*=0.04), decitabine (*r*_p_=−0.29; *P*=0.04) and gemcitabine (*r*_p_=−0.33; *P*=0.02). Deoxycytidine kinase (*dCK)* and cytidine deaminase (*CDA*) mRNA expression seemed to correlate with *in vitro* sensitivity to gemcitabine (*r*_p_=−0.31; *P*=0.03) and decitabine (*r*_p_=0.33; *P*=0.03), respectively. The *dCK/PN-I* ratio correlated inversely with LC_50_ values for gemcitabine (*r*_p_=−0.45, *P*=0.001) and the *dCK/CDA* ratio seemed to correlate with LC_50_ values for decitabine (*r*_p_=−0.29; 0.04). In conclusion, decreased expression of *hENT1*, which transports ara-C across the cell membrane, appears to be a major factor in ara-C resistance in childhood AML.

Although the treatment of acute leukaemia has improved significantly over the past few decades, the prognosis of acute myeloid leukaemia (AML) remains relatively poor. For newly diagnosed patients, the compete remission (CR) rate reaches 85–90% with standard induction chemotherapy (Creutzig *et al*, 1999; Hann *et al*, 2004). However, about 30–50% of the patients that achieve CR relapse from minimal residual disease cells that apparently survived chemotherapy (Hann *et al*, 2004), giving rise to a more resistant leukaemia. Resistance to chemotherapy therefore remains a major obstacle in the treatment of AML.

In combination with anthracyclines, 1-*β*-D-arabinofuranosylcytosine (cytosine arabinoside, cytarabine, ara-C) is the most effective agent for the treatment of AML. Ara-C is a deoxynucleoside analogue that has to be converted into its active triphosphate derivative (ara-CTP) to exert its cytotoxic effect (Grant, 1998). Ara-CTP is then incorporated into the DNA causing chain termination, resulting in a block in DNA synthesis and facilitating programmed cell death (Figure 1). Ara-C is a hydrophilic molecule and as such requires facilitated diffusion via nucleoside-specific membrane transport carriers to enter cells (Cass *et al*, 1998; Clarke *et al*, 2002). The human equilibrative nucleoside transporter (hENT1) is responsible for 80% of ara-C influx in human leukemic blast cells (Sundaram *et al*, 2001; Clarke *et al*, 2002). Inside the cell, conversion of ara-C into ara-CMP by deoxycytidine kinase (dCK) is believed to be the rate-limiting step in the metabolism of ara-C (Liliemark *et al*, 1985; Plunkett *et al*, 1987). Subsequently, ara-CMP is phosphorylated into ara-CDP by nucleoside monophosphate kinases, which in turn finally is phosphorylated into ara-CTP by diphosphate kinases (Hande and Chabner, 1978). Inactivation of ara-C results from deamination by cytidine deaminase (CDA) (Laliberte and Momparler, 1994). In addition, ara-CMP can be dephosphorylated by pyrimidine nucleotidase I (PN-I) (Amici *et al*, 1997) as well as deaminated by deoxycytidylate deaminase (dCMPD) (Mancini and Cheng, 1983). Inactivation by these enzymes decreases the amount of ara-CTP and thereby limits ara-C mediated cytotoxicity. Deoxycytidine triphosphate (dCTP) is thought to inhibit the phosphorylation of ara-C (by feedback inhibition of dCK) and the incorporation of ara-CTP into DNA (by competition for DNA polymerase). Increased intracellular dCTP pools therefore antagonise the formation of ara-CTP (Liliemark and Plunkett, 1986; White and Capizzi, 1991). dCTP can be synthezised directly via the *de novo* pathway by ribonucleotide reductase (RR) (Smith and Karp, 2003). Ribonucleotide reductase catalyses the conversion of ribonucleotides into deoxyribonucleotides (Smith and Karp, 2003). Mammalian RR is made up of two subunits (Reichard and Ehrenberg, 1983). The M1 subunit is the binding site for nucleotides and the second subunit, M2, is a metal binding site that requires both a non-haeme iron and a tyrosine-free radical for its activity (Smith and Karp, 2003). CTP synthetase (CTPs) is responsible for the conversion of uridine triphosphate (UTP) into CTP and has a high activity in several malignancies, including acute lymphoblastic leukaemia (Verschuur *et al*, 1998).

In addition to ara-C a variety of other deoxynucleoside derivatives are active in both haematological and solid malignancies. The purine analogues 2-chlorodeoxyadenosine (cladribine; 2-CdA) and fludarabine (F-ara-A) are active against indolent lymphoid malignancies and are currently also used for the treatment of hairy-cell leukaemias and chronic and acute leukaemias, respectively (Frewin and Johnson, 2001). The pyrimidine analogue gemcitabine (dFdC) has activity in various solid malignancies and some haematological disorders (Van Moorsel *et al*, 1997). The cytidine analogue 5-aza-2′-deoxycytidine (decitabine; DAC) is a potent hypomethylating agent and has shown to be active in the treatment of haematological malignancies such as AML, chronic myeloid leukaemia (CML) and myelodysplastic syndrome (Lyons *et al*, 2003). These compounds are activated intracellularly via the same metabolic pathway as ara-C. Impaired transport decreased activation of deoxynucleoside analogues to their cytotoxic tri-phosphate form or increased dCTP levels may result in resistance to this clinically important group of compounds. The objective of our study was to identify possible mechanisms of resistance to deoxnucleoside analogues, particularly ara-C, in the leukemic blasts of paediatric AML patients. We therefore determined the mRNA level of the following targets: *hENT1*, *dCK*, *PN-I*, *CDA*, *dCMPD*, *CTP* synthetase and *RR* (subunit 1 and 2) in leukaemic blasts from children with newly diagnosed AML. In addition, we studied the mRNA expression levels of the target enzymes in different AML FAB-type subgroups. Finally, the expression levels of the above-mentioned enzymes were correlated to *in vitro* sensitivity to deoxynucleoside analogues (ara-C, 2-CdA, DAC, F-ara-A and dFdC).

## MATERIALS AND METHODS

### Patient samples

Bone marrow and/or peripheral blood samples were collected from untreated children diagnosed with *de novo* AML. The following groups participated in this study and provided patient samples: (1) The Dutch Childhood Oncology Group (DCOG), The Hague, The Netherlands; (2) MRC Childhood Leukaemia Working Party, UK and (3) The AML BFM-study Group, Münster, Germany. Central review of the diagnosis, data collection as well as review of FAB-classification were carried out by reference laboratories and data centres of these groups. The FAB-classification was performed according to the criteria by Bennett *et al* (1985), including the modifications to diagnose FAB M0 and FAB M7. Samples were collected at the VU university medical centre between October 1990 and September 2002.

### Treatment protocols

Patients were treated on intensive ara-C/anthracyclines based protocols in the Netherlands, Germany and the UK (protocols DCOG AML 87 and 97, BFM 93 and 98 and MRC AML 12). The treatment protocols have been reported in detail elsewhere (Creutzig *et al*, 1995, 1999; Hann *et al*, 2004; Slats *et al*, 2005).

In the AML BFM 93 study, the patients were stratified according to risk group. At diagnosis, patients were randomised between daunorubicin (plus ara-C and etoposide, ADE) and idarubicin (plus ara-C and etoposide, AIE) induction therapy. For HR patients, one of the intensification blocks was changed to high-dose ara-C with mitoxantrone (HAM). Sibling SCT was advised for HR patients in first CR. SR patients did not recieve HAM. Protocol AML BFM 98 consisted of induction with the idarubicin block, followed by HAM. In the consolidation phase, patients were randomised for either recieving the 6-week consolidation block followed by one intensification block *vs* three intensive courses of chemotherapy.

The DCOG AML 87 protocol was based on the concurrent AML-BFM protocol. In brief, DCOG AML 87 started with an 8-day induction course followed by a 6-week consolidation block. Then two intensification courses were given. Intrathecal chemotherapy was given as central nervous system prophylaxis. Contrary to the AML BFM 87 study, no maintenance therapy was given. Sibling donor allogeneic STC was advised for HR patients in first CR.

Patients enrolled in the DCOG AML 97, which was identical to the MRC AML12 protocol, were stratified according to cytogenetics. Good risk patients (defined as patients with t(8;21), inv(16) or t(15;17)) were not eligible for SCT. Patients were randomised to induction treatment with either ADE (ara-C, daunorubicin and etoposide) or MAE (mitoxantrone, ara-C and etoposide), followed by a four or five (randomised) treatment courses. The fifth course was high-dose ara-C and asparaginase. If a matched sibling donor was available, then SCT was recommended as the fourth or fifth course (randomised).

### Cells

Mononuclear cells were isolated by density gradient centrifugation using Lymphoprep (density 1.077 g ml^−1^; Nycomed Pharma, Oslo, Norway), and centrifuged at 480 **g** for 15 min at room temperature. Cells were washed and resuspended in culture medium consisting of RPMI 1640 medium (Dutch modification without L-glutamine; Gibco BRL, Life Technologies, Breda, The Netherlands), 20% fetal calf serum (FCS; Integro, Zaandam, The Netherlands), 2 mM L-glutamine (Gibco BRL, Life Technologies), 5 *μ*g ml^−1^ transferrin, 5 ng ml^−1^ sodium selenite (ITS media supplement; Sigma, St Louis, MO, USA), 100 IU ml^−1^ penicillin, 100 *μ*g ml^−1^ streptomycin, 0.125 *μ*g ml^−1^ fungizone (Gibco BRL, Life technologies), and 0.2 mg ml^−1^ gentamycin (Gibco BRL, Life technologies). Contaminating normal cells were removed by immunomagnetic beads (in case of lymphocytes) or by freezing in liquid nitrogen and thawing (in case of granulocytes) (Kaspers *et al*, 1994b). All samples contained at least 80% leukemic cells, as determined morphologically on May–Grunwald–Giemsa (Merck, Darmstadt, Germany) stained cytospins. A minimum of 5 × 10^6^ cells were lysed in RNAzol or Trizol reagent (Gibco BRL, Life technologies) and stored at −80°C until RNA extraction. The majority of samples were received and processed within 24 h (*n*=42), eight samples however were received and processed within 48 h.

### RNA extraction and cDNA synthesis

Total cellular RNA was isolated from 5 × 10^6^ cells using RNAzol or Trizol reagent, according to the manufacturer's protocol. After precipitation with ethanol, RNA pellets were dissolved in water. The RNA was quantitated spectrophotometrically. cDNA synthesis was performed as described by Stam *et al* (2003). Briefly, following a denaturation step of 5 min at 70°C, 1 *μ*g of RNA was reverse transcribed to single-stranded cDNA using a mix of random hexamers (2.5 *μ*M) and oligo dT primers (20 nM). The RT reaction was performed in a total volume of 25 *μ*l containing 0.2 mM of each dNTP (Amersham Pharmacia, Biotech, Piscataway, NJ, USA) 200 U Moloney murine leukaemia virus reverse transcriptase (M-ML RT; Promega, Madison, WI, USA), and 25 U RNAsin (Promega) at 37°C for 30 min, 42°C for 15 min and 94°C for 5 min. The obtained cDNA was diluted to a final concentration of 8 ng *μ*l^−1^. Samples were stored at −80°C.

### Quantitative real-time PCR (Taqman technology)

The mRNA expression levels of *dCK*, *PN-I*, *CDA*, *dCMPD*, *hENT1*, *RR1* and *RR2*, *CTP* synthetase and the endogenous housekeeping gene encoding glyceraldehyde-3-phosphate dehydrogenase (*GAPDH*) as a reference were quantified using real-time PCR analysis (Taqman) on an ABI Prism 7700 sequence detection system (PE Applied Biosystems). Amplification of specific PCR products was detected using dual-fluorescent nonextendable probes labelled with 6-carboxyfluorescein (FAM) at the 5′ end and with 6-carboxytetramethylrhodamine (TAMRA) at the 3′ end. All primers and probe combinations were designed using the OLIGO 6.22 software (Molecular Biology Insights, Cascade, CO, USA) and purchased from Eurogentec (Seraing, Belgium). Primers and probes used to detect *hENT1, dCK, PN-I, CDA* and *dCMPD* have been reported before (Stam *et al*, 2003). For *CTPs* and *RR1* and *RR2* primers and probes are listed in Table 1.

As described before (Stam *et al*, 2003), real-time PCR was performed in a total reaction volume of 50 *μ*l containing TaqMan buffer A (Applied Biosystems, Nieuwerkerk a/d Ijssel, The Netherlands), 4 mM MgCl_2_, 200 *μ*M of each dNTP (Amersham Pharmacia Biotech), 300 nM forward and reverse primer, 50 nM dual-labelled fluorogenic internal probe, 1.25 U Ampli*Taq* Gold DNA polymerase (Applied Biosystems) and 40 ng of cDNA as a template. Samples were heated for 10 min at 95°C to activate the Ampli*Taq* Gold DNA polymerase and amplified during 40 cycles of 15 s at 95°C and 60 s at 60°C. The relative mRNA expression levels of the target genes in each patient were calculated using the comparative cycle time (*C*_t_) method (Meijerink *et al*, 2001). Briefly, this PCR *C*_t_ value is the cycle number at which emitted fluorescence exceeds 10 × the standard deviation (s.d.) of baseline emissions as measured from cycles 3–15. The *C*_t_ of the target gene is normalised to the GAPDH PCR *C*_t_ value by subtracting the GAPDH *C*_t_ value from the target *C*_t_ value. The mRNA expression level for each target PCR relative to *GAPDH* can was calculated using the following equation: 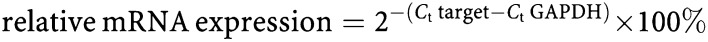


### *In vitro* cytotoxicity assay

*In vitro* cytotoxicity of the deoxynucleoside analogues ara-C (Cytosar; Pharmacia &Upjohn, Woerden, The Netherlands), 2-CdA (Leustatin, Ortho Biotech, USA), DAC (Decitabine, kindly provided by PCH Pharmachemie bv, Haarlem, The Netherlands), F-ara-A (Fludara, Schering AG, The Netherlands), Gemcitabine (Gemzar, Eli Lilly, Houten, The Netherlands) was determined using the MTT-assay as described previously (Pieters *et al*, 1990). Briefly, cells were cultured in round-bottomed 96-well microtitre plates in the presence of six concentrations of different drugs, in the following ranges: ara-C (0.04–41.0 *μ*M); 2-CdA (0.001–140.0 *μ*M); DAC (11.0 *μ*M–11.0 mM); F-ara-A (0.04–44.0 *μ*M) and dFdC (0.04–13.0 mM). Cells without drugs were included as controls and cells in culture medium only were used as blanks. The plates were cultured for 4 days at 37°C in humidified air containing 5% CO_2_, after which 10 *μ*l of 3-[4,5-dimethylthiazol-2-yl]-2,5 diphenyl tetrazoliumbromide (MTT; 5 mg ml^−1^, Sigma Aldrich, Zwijndrecht, The Netherlands) was added and the plates were incubated for an additional 6 h. Only viable cells are able to reduce MTT tetrazolium salt to purple/blue formazan crystals. The formazan crystals were dissolved using acidified isopropanol (0.04 N HCl-isopropyl alcohol) and the optical density (OD), which is linearly related to the number of viable cells, (Klumper *et al*, 1995) was measured spectophotometrically at 562 and 720 nM. After subtraction of the blank values, the leukemic cell survival (LCS) was calculated by the following equation: LCS=(OD_day4_ treated well/mean OD_day4_ control wells) × 100%. Drug sensitivity was expressed as the LC_50_ value, the drug concentration lethal to 50% of the leukemic cells. Evaluable results were obtained when a minimum of 70% leukemic cells was present at day 4 and when the control OD was more than or equal to 0.050 (Kaspers *et al*, 1994a). Sample source (bone marrow or peripheral blood) and cryo-preservation do not influence the results obtained by cellular drug resistance testing and were therefore analysed together (Kaspers *et al*, 1991).

### Statistics

Distribution of measurement values was characterised with median and quartiles (25th–75th percentiles). Due to the strongly skewed character of the distributions, analyses were performed on the log-transformed measurements. For significance, a two-tailed level of *α*=0.01 was used. *P*-values between 0.01 and 0.05 were considered to indicate a trend for significance. Pearson correlations were used to describe relations between variables. AML patient samples were divided in thre subgroups, according to their sensitivity to ara-C: sensitive (LC_50_<0.98 *μ*M), intermediate (0.98<LC_50_<5.18 *μ*M) and resistant (LC_50_>5.18 *μ*M) (cutoffs based on Zwaan ChM, *Blood*, 2000) (Zwaan *et al*, 2000) and a one-way ANOVA was carried out on *hENT1* for these three sensitivity groups. Stepwise modelling on the log-transformed LC_50_ values was used to unravel the relative importance of the possible indicators.

## RESULTS

### Patient characteristics

Fifty-five AML patient samples with LC_50_ values for ara-C, cladribine, decitabine, fludarabine and gemcitabine (determined with the MTT assay) were selected for RNA isolation. We were unable to isolate a sufficient amount of RNA from five of these samples. Thus, the study population consists of 50 newly diagnosed paediatric AML patients. Patient characteristics are listed in Table 2. This selected group of AML patients did not differ significantly with regard to age (*P*=0.30), WBC (*P*=0.14), sex (*P*=0.39) or *in vitro* sensitivity to ara-C (*P*=0.50) from a large group of AML samples that we have previously characterised for *in vitro* drug sensitivity (Hubeek *et al*, submitted; Zwaan *et al*, 2000) and was therefore considered to be representative.

### mRNA expression levels of enzymes involved in the metabolism of deoxynucleoside analogues in AML and FAB-type subgroups

**Figure 2:**
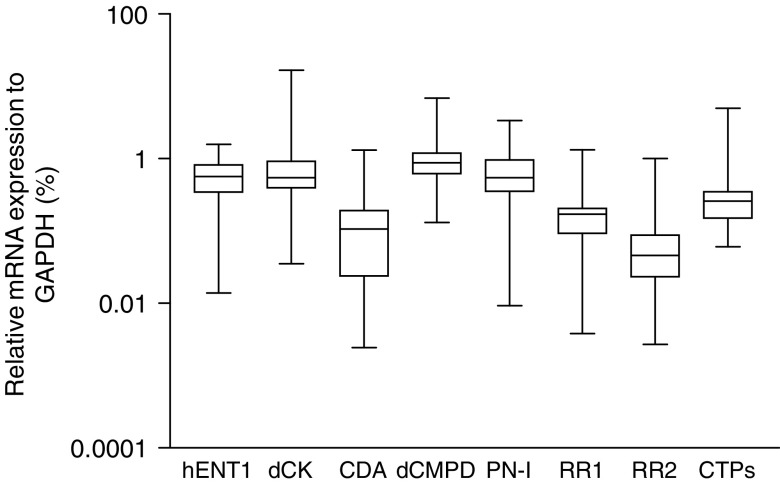
Expression levels of potential resistance factors to ara-C, determined by Taqman PCR. mRNA expression (relative to glyceraldehyde-3-phosphate dehydrogenase (*GAPDH*)) of the human equilibrative nucleoside transporter 1 (*hENT1*), deoxycytidine kinase (*dCK*), pyrimidine nucleotidase I (*PN-I*), cytidine deaminase (*CDA*), ribonucleotide reductase subunit 1 and 2 (*RR1* & *RR2*) and CTP synthetase (*CTPs*) were determined by Taqman PCR in 50 paediatric AML samples, obtained at diagnosis. Measurable amounts of all genes were found in all patients. Targets were expressed with great variability; group medians, 25th/75th percentiles and the ranges are given.

Using quantitative real-time PCR the mRNA expression levels of *hENT1, dCK, PN-I, CDA, dCMPd, RR1, RR2* and *CTPs* were determined. Measurable amounts of all eight genes were found in all samples. Sample source (bone marrow (*n*=37) or peripheral blood (*n*=13)) and the time interval between tissue acquisition and processing/storage of the cells (within 24 or 48 h) did not influence mRNA expression of the enzymes and all samples were therefore evaluated together in the following analyses. Genes were expressed with considerable variability between various patients (Figure 2). We investigated the association between all eight genes and several diagnostic features. There was no difference in mRNA expression levels of target genes between boys and girls, nor was there a relation between the expression level of these genes and initial white blood cell (WBC) count.

For the analysis with FAB-type, patients were divided into three subgroups: FAB M1/M2, FAB M4 and FAB M5. FAB M1/M2 were taken together because they did not differ in age, sex, WBC, drug resistance or mRNA expression levels (data not shown). FAB M0 and FAB M3 were excluded because of the limited number of samples. FAB M5 expressed 1.8-fold (*P*=0.007) lower levels of *PN-I* compared to FAB M1/M2. We did not observe any other significant differences (Table 3).

### *In vitro* cytotoxicity assay

Dose–response curves were obtained for all drugs and marked differences between individual patients were found. The median (25th–75th percentile) ara-C LC_50_ value was 1.70 *μ*M (0.59–3.38 *μ*M; *n*=50), which is in concordance with results published previously (Zwaan *et al*, 2000). For the purine analogues 2-CdA and F-ara-A group median LC_50_ values were 0.073 *μ*M (0.051–0.098 *μ*M; *n*=46) and 1.19 *μ*M (0.66–2.27 *μ*M; *n*=47), respectively. The group median for dFdC was 10.04 *μ*M (2.05–20.86 *μ*M; *n*=48), while DAC was only active in very high concentrations (median LC_50_ value=3426 *μ*M (717–5700 *μ*M; *n*=48)).

### Correlations between mRNA expression levels and *in vitro* sensitivity to deoxynucleoside analogues

*hENT1* mRNA expression inversely correlated with the LC_50_ values of ara-C (*r*_p_=−0.46; *P*=0.001; *n*=50) and also seemed to correlate inversely with the LC_50_ values of 2-CdA (*r*_p_=−0.30; *P*=0.04; *n*=46), DAC (*r*_p_=−0.29; *P*=0.04; *n*=48) and dFdC (*r*_p_=−0.33; *P*=0.02; *n*=48). In other words, increased sensitivity to deoxynucleoside analogues was directly related to increased mRNA expression of the *hENT1* nucleoside transporter. Furthermore, decreased *dCK* mRNA expression seemed to correlate with resistance to dFdC (*r*_p_=−0.31; *P*=0.03; *n*=48). Also, resistance to DAC seemed to correlate with increased *CDA* mRNA levels (*r*_p_=0.33; *P*=0.03; *n*=48). The accumulation of ara-CTP could depend on the ratio of the activation enzyme dCK and the inactivation enzymes *PN-I* and *CDA*. Therefore, we also studied the relation between the *dCK/PN-I* and *dCK/CDA* ratios and *in vitro* drug sensitivity. The *dCK/PN-I* ratio correlated inversely with the LC_50_ values for dFdC (*r*_p_=−0.45; *P*=0.001; *n*=47) and the *dCK/CDA* ratio seemed to correlate with the LC_50_ values for DAC (*r*_p_=−0.29; 0.04; *n*=48). We did not observe correlations between these ratios and *in vitro* sensitivity to ara-C, 2-CdA and F-ara (Table 4).

**Figure 3:**
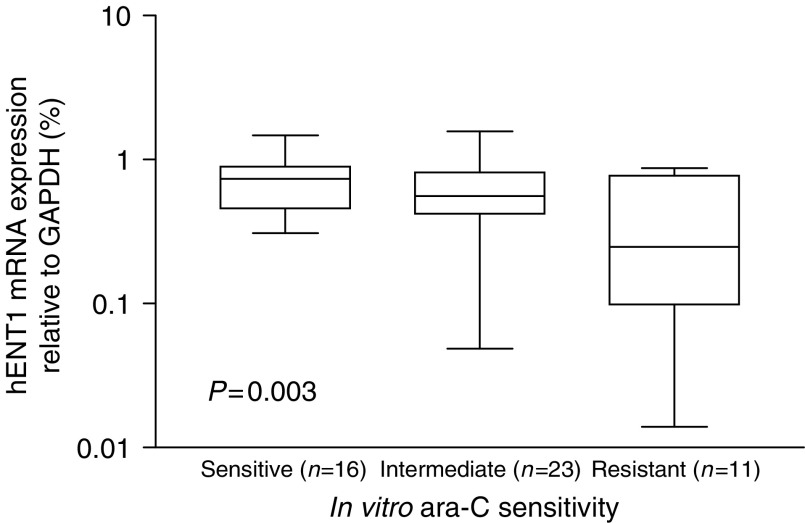
*hENT1* mRNA expression in relation to *in vitro* ara-C sensitivity. AML patient samples were divided into three groups based on *in vitro* ara-C sensitivity (sensitive=LC_50_<0.98 *μ*M), intermediate=0.98<LC_50_ <5.18 *μ*M) and resistant=LC_50_>5.18 *μ*M) (Zwaan *et al*, 2000). Patient resistant to ara-C *in vitro* expressed 3.0-fold lower hENT1 mRNA levels compared to sensitive patients. The lines indicate the median value. *P*-value determined by one-way ANOVA.

mRNA expression levels of *hENT1, dCK, PN-I, CDA, dCMPd, RR1/2* and *CTPs* were entered into a stepwise multivariate regression model to identify the most important indicators with respect to *in vitro* sensitivity to deoxynucleoside analogues (dependent variables LC_50_ values ara-C, 2-CdA, DAC, F-ara-A or dFdC). In multivariate analysis, *hENT1* mRNA expression predicted *in vitro* sensitivity to ara-C (*P*=0.002). Furthermore, *CDA* mRNA expression levels seemed to predict *in vitro* sensitivity to DAC (*P*=0.02), while other factors did not reach significance. Also, when we divided the AML samples in three subgroups based on their *in vitro* ara-C sensitivity, resistant patients expressed three-fold lower *hENT1* mRNA levels compared to sensitive patients (*P*=0.003; Figure 3).

## DISCUSSSION

For AML, ara-C is the essential drug during induction and consolidation therapy and is given both at intermediate and high-dose schedules (Bloomfield *et al*, 1998; Galmarini *et al*, 2002a). In the present study we analysed possible resistance factors to ara-C, and other clinically important deoxynucleoside analogues, in AML by measuring the gene expression of the major players in transport and metabolism of ara-C. Quantitative real-time PCR revealed that AML FAB-M5 expressed lower levels of the ara-C degrading enzyme *PN-I* compared to FAB-M1/2. Although the analysis included only a limited number of samples, this finding may provide an explanation for the relative sensitivity to ara-C of AML FAB-M5 that we reported previously (Zwaan *et al*, 2000).

We studied the relation between the mRNA expression level of potential ara-C resistance factors and *in vitro* sensitivity to deoxynucleoside analogues. Although *in vitro* drug resistance testing differs considerably from the *in vivo* situation, it does provide valuable indications as to which factors might be important in drug sensitivity *in vivo* (Kaspers *et al*, 1999). *hENT1* mRNA expression correlated with sensitivity to ara-C and also seemed to correlate with sensitivity to 2-CdA, DAC and dFdC, indicating that transport across the cell membrane is an important step for deoxynucleoside analogue induced cytotoxicity. In multivariate analysis, *hENT1* mRNA expression was the most important factor determining sensitivity to ara-C. This might be explained by the fact that entry of ara-C into the cell is mainly dependent on hENT1-mediated transport (Wiley *et al*, 1982; Pastor-Anglada *et al*, 1998; Sundaram *et al*, 2001; Clarke *et al*, 2002). In contrast, 2-CdA, DAC and dFdC differ from ara-C with respect to their preferential nucleoside transporters and can be transported across the cell membrane by other members of the nucleoside transporter family as well (Damaraju *et al*, 2003). 2-CdA can enter cells via hENT1, hENT2 and the human concentrative nucleoside transporter (hCNT) 3 (Ritzel *et al*, 2001; Damaraju *et al*, 2003), while hENT1, hENT2, hCNT1 and hCNT3 are able to mediate uptake of dFdC into cells (Damaraju *et al*, 2003). hENT1 mediated influx however seems to be a pivotal factor in ara-C cytotoxicity. Patients resistant to ara-C *in vitro* expressed three-fold lower *hENT1* mRNA levels. Our results are supported by the fact that hENT1 has been implicated as a crucial factor in ara-C sensitivity in previous studies (Galmarini *et al*, 2002b, 2002c). Galmarini *et al* measured *hENT1* mRNA expression in adult AML samples and demonstrated that *hENT1* deficiency was related to a shorter disease-free survival (Galmarini *et al*, 2002c). In addition, elevated *hENT1* mRNA expression explained the remarkable ara-C sensitivity of infants with *MLL* gene-rearranged ALL (Stam *et al*, 2003). *hENT1* may therefore be a valuable predictor of ara-C sensitivity at diagnosis. Unfortunately, we were not able to asses the relation between *hENT1* expression and *in vivo* response to treatment due to the heterogenity of the treatment and the limited follow-up time. Patients were treated according to different treatment protocols and several different dosing schedules. AML patients may however benefit from screening for *hENT1* mRNA levels at diagnosis, because of its significance for ara-C dosing. At intermediate dose ara-C (100–200 mg m^−2^) plasma levels are in the *μ*M range and transport across the cell membrane is solely dependent on nucleoside transporters (Peters *et al*, 1993). At high-dose ara-C (1–3 g m^−2^), however, hENT1 seems less crucial although plasma concentrations might not exceed the Km of the transporter mediated influx. Ara-C may also enter by passive diffusion at this concentration, while dCK will be saturated (Capizzi *et al*, 1985; Bolwell *et al*, 1988). Patients with a low *hENT1* mRNA level could potentially benefit from up-front high dose ara-C treatment or an ara-C analogue that is not dependent on transporter-mediated influx. A compound such as troxacitabine, which passively diffuses across the cell membrane due to its unusual L-configuration (Gourdeau *et al*, 2001; Giles, 2002), might be able to circumvent ara-C resistance caused by low *hENT1* expression.

Most studies on ara-C resistance have focussed on the conversion of ara-C to ara-CTP and several studies have linked reduced *dCK* mRNA expression or functional activity to ara-C resistance (Owens *et al*, 1992; Stegmann *et al*, 1993; Kawasaki *et al*, 1996; Dumontet *et al*, 1999). In acute leukaemia, relapsed ALL and AML patients have been shown to express decreased dCK mRNA levels (Kakihara *et al*, 1998). In contrast, dCK was not rate-limiting in the formation of ara-CTP in infants with *MLL* gene-rearranged ALL (Stam *et al*, 2003). In this present study, we did not observe a correlation between *dCK* mRNA expression and *in vitro* ara-C sensitivity in AML blasts. Although there was a considerable range in *dCK* mRNA levels in AML blasts, the median expression was quite high, and it therefore seems unlikely that low dCK expression plays a role in ara-C resistance in childhood AML at diagnosis. We have previously reported on *dCK* mRNA levels in childhood AML blasts and most AML samples expressed mRNA leves that were in the range of cell lines sensitive to ara-C (van der Wilt *et al*, 2003). Reduced *dCK* mRNA expression may however be involved in resistance to gemcitabine. Both *dCK* mRNA and protein levels have been shown to predict *in vivo* gemcitabine sensitivity (Kroep *et al*, 2002). Our present study also indicated that reduced *dCK* mRNA expression may contribute to *in vitro* gemcitabine resistance in AML blasts. It should be mentioned, however, that the Pearson correlation coefficient was low and was not significant in multivariate analysis.

Finally, multivariate analysis showed that DAC resistance seemed to correlate with increased mRNA levels of the inactivating enzyme CDA. DAC was initially developed as a cytotoxic agent and has activity in several haematological malignancies. Low-dose DAC is currently enjoying a revival as a specific inhibitor of DNA hypermethylation in cancer (Lyons *et al*, 2003). DAC is an excellent substrate for CDA and elevated *CDA* mRNA levels might contribute to resistance to DAC (Momparler, 1985).

In conclusion, our findings indicate that reduced drug influx into the cell caused by decreased *hENT1* mRNA expression might be involved in resistance to ara-C, and other deoxynucleoside analogues, in childhood AML.

## Figures and Tables

**Figure 1:**
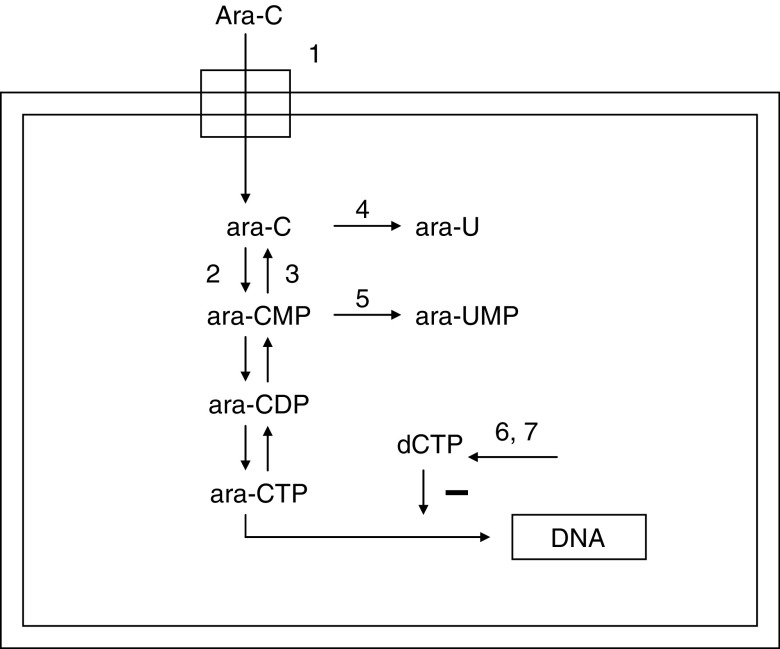
Metabolism of ara-C. Ara-C enters the cell via the equilibrative nucleoside transporter 1 (hENT1; 1). Inside the cell, ara-C is phosphorylated to ara-CMP by deoxycytidine kinase (dCK; 2). Ara-CMP is subsequently phosphorylated to ara-CTP, the active metabolite. Incorporation of ara-CTP into the DNA blocks DNA synthesis and leads to cell death. Ara-CTP formation can be obstructed. Pyrimidine nucleotidase I (PN-I; 3) opposes the action of dCK. Cytidine deaminase (CDA; 4) and deoxycytidylate deaminase (dCMPD; 5) convert ara-C to ara-U, and ara-CMP to ara-UMP, respectively. Increased intracellular dCTP pools antagonise the formation of ara-CTP. dCTP can be synthesised directly via the *de novo* pathway by ribonucleotide reductase (6). CTP synthetase (CTPs; 7) converts uridine triphosphate to CTP. Because aberrant expression of these enzymes may be related to *in vitro* sensitivity to ara-C, and other deoxynucleoside analogues, we determined the mRNA expression of the target genes in AML.

**Table 1:** Primers and probe combinations used for the quantitative real-time PCR

**Gene**	**Sequence**
*RR1*	
Forward	5′-GTG TGG GAA ATC TCT CAG A-3′
Reverse	5′-CCA TGG CTG CTG TGT T-3′
Probe	5′-(FAM)-CAA ACT CAC TAG TAT GCA CTT CTA CGG-(TAMRA)-3′
	
*RR2*	
Forward	5′-AGG GGC TCA GCT TGG-3′
Reverse	5′-GGG GCA GCT GCT TTA G-3′
Probe	5′-(FAM)-CGT CCT GGC CAG CAA GAC-(TAMRA)-3′
	
*CTPs*	
Forward	5′-ATC CCG TGG TCG TAG AC-3′
Reverse	5′-TGG CCA ACA AAC TTC AA-3′
Probe	5′-(FAM)-AAC ACA ACC CAG GGC AGA TG-(TAMRA)-3′

RR1=ribonucleotide reductase subunit 1; RR2=ribonucleotide reductase subunit 2; CTPs=CTP synthetase.

**Table 2:** Patient characteristics

**Patient characteristics**	***n***
Sex (male : female)	31 : 19
Age, years (median, range)	10.7 (0.1–16.8)
WBC (median, range)	79.3 × 10^9^ l^−1^ (2.1–524.0)
	
*FAB-type*	
M0	2
M1	6
M2	8
M3	4
M4	18
M5	9
Unknown	3

WBC=white blood cell count.

**Table 3:** mRNA expression (relative to *GAPDH* (%)) of *hENT1* and enzymes involved in ara-C cytotoxicity in AML FAB type subgroups

	**FAB M1/2**	**FAB M4**	**FAB M5**
	**(*n*=14)**	**(*n*=18)**	**(*n*=9)**
*hENT1*	0.68 (0.30–0.76)	0.51 (0.33–0.75)	0.57 (0.48–0.95)
*DCK*	0.61 (0.45–1.34)	0.52 (0.42–0.89)	0.55 (0.17–0.85)
*CDA*	0.069 (0.009–0.24)	0.14 (0.06–0.20)	0.16 (0.08–0.21)
*DCMPd*	1.09 (0.72–1.56)	0.89 (0.70–1.19)	0.60 (0.52–1.09)
*PN-I*	0.79 (0.51–1.37)	0.60 (0.39–0.85)	0.43 (0.16–0.50)*
*RR1*	0.18 (0.11–0.29)	0.15 (0.06–0.22)	0.15 (0.08–0.22)
*RR2*	0.05 (0.02–0.09)	0.04 (0.01–0.08)	0.05 (0.02–0.17)
*CTPs*	0.28 (0.20–0.32)	0.20 (0.14–0.33)	0.34 (0.17–0.47)

Values are the group median (25th–75th percentile).

* *P*<0.01 compared to FAB M1/2.

**Table 4:** Correlation between mRNA level of potential resistance factors and *in vitro* sensitivity to deoxynucleoside analogues (expressed as LC_50_ values) in 50 paediatric AML samples obtained at initial diagnosis

		**Ara-C**	**2-CdA**	**DAC**	**F-ara-A**	**dFdC**
*hENT1*	*r*_p_	−0.46**	−0.30*	−0.29*	−0.24	−0.38*
	*P*-value	0.001	0.04	0.04	0.09	0.02
*dCK*	*r*_p_	−0.11	−0.09	−0.04	0.004	−0.31*
	*P*-value	0.43	0.55	0.79	0.98	0.03
*CDA*	*r*_p_	0.13	−0.09	0.33*	0.06	0.001
	*P*-value	0.38	0.57	0.02	0.69	0.99
*dCK/PN-1*	*r*_p_	−0.27	−0.09	−0.16	−0.26	−0.45**
	*P*-value	0.06	0.55	0.29	0.08	0.001
*dCK/CDA*	*r*_p_	−0.17	0.02	0.29*	−0.05	−0.18
	*P*-value	0.24	0.87	0.04	0.75	0.23

** Pearson correlation (*r*_p_) significant at the 0.01 level (two-tailed).

* Pearson correlation (*r*_p_) significant at the 0.05 level (two-tailed).

Correlations between mRNA expression of *dCMPD, PN-I, RR1, RR*2 and *CTPs*, and *in vitro* sensitivity to deoxynucleoside analogues were not significant.
